# Spectral Signatures of X-ray Scatter Using Energy-Resolving Photon-Counting Detectors

**DOI:** 10.3390/s19225022

**Published:** 2019-11-18

**Authors:** Cale E. Lewis, Mini Das

**Affiliations:** Department of Physics, University of Houston, Houston, TX 77204, USA; celewis2@central.uh.edu

**Keywords:** X-ray scatter, photon-counting detectors, imaging, Compton scatter

## Abstract

Energy-resolving photon-counting detectors (PCDs) separate photons from a polychromatic X-ray source into a number of separate energy bins. This spectral information from PCDs would allow advancements in X-ray imaging, such as improving image contrast, quantitative imaging, and material identification and characterization. However, aspects like detector spectral distortions and scattered photons from the object can impede these advantages if left unaccounted for. Scattered X-ray photons act as noise in an image and reduce image contrast, thereby significantly hindering PCD utility. In this paper, we explore and outline several important characteristics of spectral X-ray scatter with examples of soft-material imaging (such as cancer imaging in mammography or explosives detection in airport security). Our results showed critical spectral signatures of scattered photons that depend on a few adjustable experimental factors. Additionally, energy bins over a large portion of the spectrum exhibit lower scatter-to-primary ratio in comparison to what would be expected when using a conventional energy-integrating detector. These important findings allow flexible choice of scatter-correction methods and energy-bin utilization when using PCDs. Our findings also propel the development of efficient spectral X-ray scatter correction methods for a wide range of PCD-based applications.

## 1. Introduction

Image contrast in transmission X-ray imaging results from attenuation variations of material types within the object. While these attenuation variations are spectrally dependent, conventional energy-integrating detectors (EIDs), widely used in clinical and industrial X-ray imaging, do not capture this important information. Significant developments in the area of energy-resolving photon-counting detectors (PCDs) are happening globally. PCDs developed by CERN Medipix collaboration [1,2] are some of the most advanced in this category. Exploiting spectral information allows quantitative material identification and characterization. Examples of these techniques include K-edge imaging [3,4,5], material decomposition [6,7,8] and phase-contrast imaging [9,10,11]. Their other advantages include zero dark noise, and the ability for flexible energy weighting [12,13,14].

Energy-resolving PCDs are direct conversion detectors. Impinging X-ray photons are converted into charge packets in the semiconducting wafer bump-bonded to a virtually pixellated electronic readout. The application-specific integrated circuit (ASIC) consists of an individual readout for each pixel. This includes an electronic discriminator to exclude photons above a given threshold energy value selected by the experimenter. Using efficient energy-calibration methods, the experimenter can accurately link these electronic thresholds to the corresponding photon energy [15].

Detector performance is highly influenced by the semiconductor sensor material. Charge-sharing limitations, such as charge diffusion and X-ray fluorescence, reduce the spectral resolution by falsely attributing a single-incident photon as one or more lower-energy photons. These distortions restrict detector design since influence increases for thicker sensors and smaller pixel sizes. High-Z materials (such as CdTe and CdZnTe [16]) have desirable X-ray absorption efficiency, but are often prone to spectral distortions resulting from K-fluorescence and polarization. These issues are diminished for low-Z material (such as silicon) at the cost of reduced absorption efficiency. In addition, charge-sharing correction techniques are being developed to improve the spectral response of PCDs [17,18]. Advanced PCDs, such as Medipix3RX (a recent version developed by the Medipix collaboration), benefit from interpixel communication known as charge-summing mode [1] while maintaining one of the highest spatial resolutions (55 μm) and multiple energy-threshold capabilities.

Inaccurate spectral-intensity measurements also occur due to photons scattered from the object. The spectral characteristics of scattered X-rays differ from the primary due to the energy transfer and angular deflection of the collision. Maintaining low scatter-to-primary ratios (SPRs) is crucial for spectral imaging where scatter degrades spectral fidelity and hence material identification [19,20], including material decomposition [21] and iodine K-edge imaging [22]. Thus, these photons generally increase the total apparent counts and decrease the contrast-to-noise ratio [23].

Here, we report on the spectral characteristics of incoherent X-ray scatter in transmission X-ray imaging using an energy-resolving PCD. We used both Monte Carlo simulations and bench-top experiments to identify key features. As a specific application example, we considered spectral and object parameters relevant to applications such as mammography. Identifying spectral and detector features that vary the SPR can help develop better PCD imaging design and scatter-correction techniques. We use both simulations and experiments to explore these questions.

## 2. Materials and Methods

### 2.1. Monte Carlo Simulations

**Figure 1:**
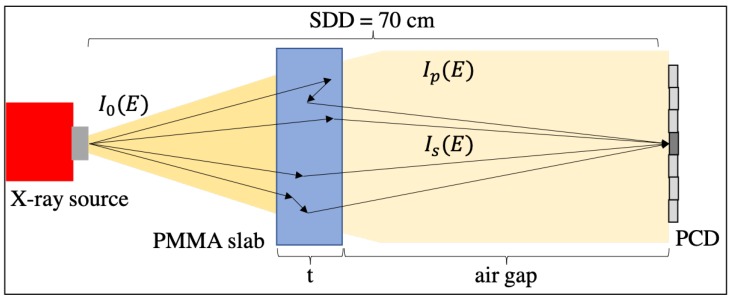
Schematic of experiment configuration.

**Figure 2:**
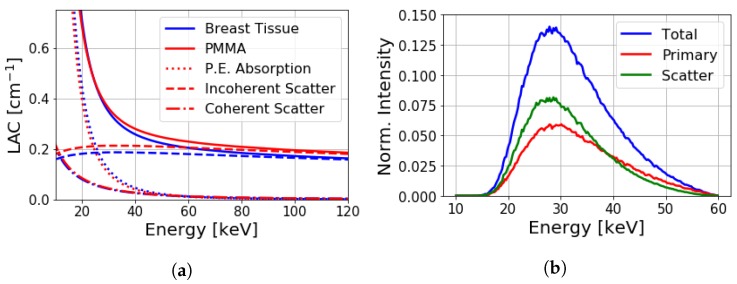
(**a**) Linear attenuation coefficients of breast tissue (blue) and polymethyl methacrylate (PMMA; red). Total linear-attenuation coefficient shown (solid curves) along with photoelectric absorption (dotted curves) and incoherent scatter components (dashed curves). (**b**) Simulated spectral distribution of total, primary, and scattered intensities for a 6.0 cm thick PMMA slab at a 0.5 cm air gap with 60 kVp incidence. Each distribution is normalized by the unattenuated source intensity.

The BEAMnrc software package [24] was used for Monte Carlo simulations to emulate our bench-top experiment (Figure 1). A tungsten anode X-ray tube was used to generate a polychromatic beam from a point source with 5 × 10${}^{7}$ X-rays per exposure. The spectral distribution of the cone-beam source was modeled by simulating the bremsstrahlung radiation of an electron-beam incidence on a tungsten target. Biological samples (and other low-Z materials like explosives) are considerably susceptible to object scatter, as demonstrated by linear-attenuation coefficients shown in Figure 2a; certain plastics like polymethyl methacrylate (PMMA) mimic their X-ray absorption and scattering properties (see Figure 2a).

In our study, X-ray photons were incident on a homogeneous PMMA slab with lateral dimensions of 12.4 × 12.4 cm${}^{2}$, and thickness range was 2.0–8.0 cm to approximate the typical breast area and thickness observed in mammography [25]. Photoelectric absorption, Rayleigh and Compton scattering (with cross-sections for bound electrons obtained from the NIST database [26,27]) were considered as predominant interactions. Photons that interacted with the PMMA were ‘tagged’ such that scatter and primary intensities could be differentiated at the detector. Figure 2b shows the simulated spectral distributions of total, primary, and scattered photons through a 6.0 cm thick PMMA slab at a 0.5 cm air gap. Under these conditions, primary and scattered photons contribute in nearly equal amounts to total measured intensity. The scattered radiation also shifted the total distribution to slightly lower energies, reflecting a spectrally dependent SPR.

Detection was performed by recording the energy of each X-ray photon that crossed the scoring region. This region was centered on the transmission axis 70 cm from the source with an area of 12.4 × 12.4 cm${}^{2}$. The proportion of scattered photons reaching the detector was altered by changing the object-to-detector distance from 0.5 to 30 cm. Spectral distributions of the detected photons were obtained with histograms ranging from 10 keV to the source peak voltage (kVp) divided into 200 bins. The resultant spectra were then modified by the absorption function of a 300 μm thick silicon detector.

Quantitative accuracy due to changing scatter counts was evaluated by estimating the linear-attenuation coefficient of the PMMA slabs under varying conditions:

(1) $$\mu \left(E\right)=-\ln\left(\frac{I(E)}{I_{0}\left(E\right)}\right)/t,$$

where I(E) and $I_{0}\left(E\right)$ are measured intensities with and without the object, respectively, and *t* is slab thickness. This was compared against the ideal (obtained from the NIST XCOM database [28]) or known attenuation values to obtain the percent error:

(2) $$\%Error=\frac{\mu \left(E\right)-\mu {\left(E\right)}^{ideal}}{\mu {\left(E\right)}^{ideal}}.$$

Finally, an EID detection scheme was approximated by summing photon counts across the spectral distributions with weights corresponding to their respective energies,

(3) $$I_{EID}=\int_{TH}^{kVp}E\cdot I\left(E\right)D\left(E\right)dE,$$

where I(E) is photon-intensity distribution, and summation ranged from the lowest photon energy at TH up to the peak photon energy (kVp). Factor $D\left(E\right)=1-e^{-\mu_{d}\left(E\right)t_{d}}$ represents detector absorption function that depends on detector-material attenuation ($\mu_{d}$) and thickness ($t_{d}$). Lower energy limit for the threshold in Equation (3) was set to 10 keV, which includes the full width of the simulated spectral distribution of the source.

### 2.2. Bench-Top Experiments

Experiments were conducted on a bench-top X-ray imaging system to validate the simulation results and to gain additional insights. X-rays were generated using a Hamamatsu microfocus X-ray tube unit (L8122-01) consisting of a tungsten anode target and a 200 μm thick beryllium output window. The tube was operated at 60 kVp with a 450 μA current and 50 μm focal spot to produce the source X-ray distribution. X-rays were propagated through rectangular PMMA slabs and recorded with a Medipix3RX PCD operated in charge-summing mode at a fixed distance (70 cm) from the source, as in the case of simulations described above.

## 3. Results

**Figure 3:**
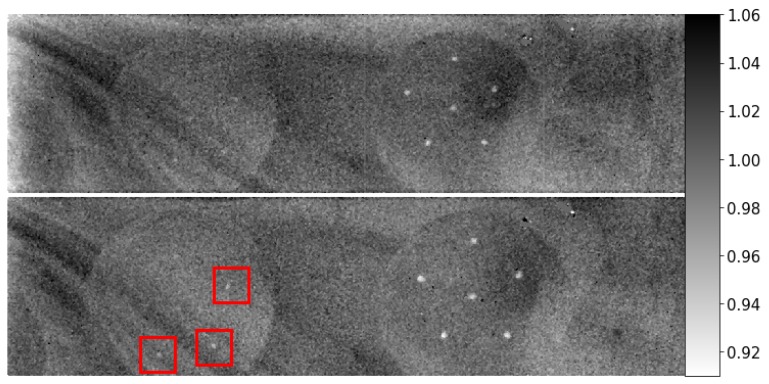
Projections of a CIRS breast phantom with embedded microcalcifications obtained at air gaps of (**top**) 1.2 cm and (**bottom**) 10.0 cm. Data for 20–30 keV energy bin.

Contrast loss due to increased scatter is demonstrated with experimentally obtained projections of a breast phantom in Figure 3. Both lesion sharpness and microcalcification contrast improved by reducing the scatter through increasing the air gap between object and detector from 1.2 to 10.0 cm.

### 3.1. Energy-Integrated SPR

**Figure 4:**
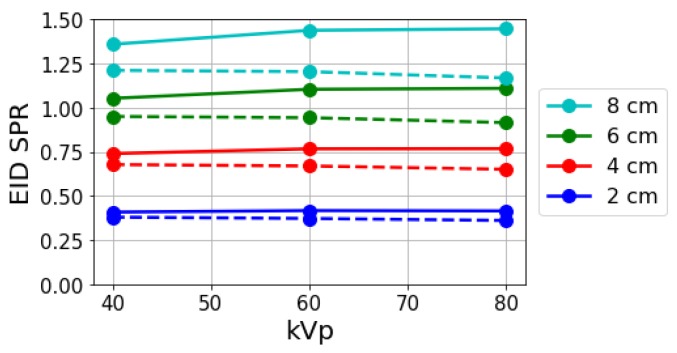
Simulated scatter-to-primary ratio (SPR) values using (solid) a 300 μm thick silicon energy-integrating detector (EID) and (dashed) an ideal EID as a function of kVp for different object thicknesses at a fixed 0.5 cm air gap.

As shown in Equation (3), photons were weighed by their energy in an EID, and all spectral information was lost in the detection process. Scattered and primary photons could easily be separated in a Monte Carlo simulation. Figure 4 shows the simulated SPR values obtained with energy-integrating detection for different kVps and object thicknesses. SPR values are provided for both a 300 μm thick silicon detector and a fully absorbing ideal detector to illustrate dependence on the detection-absorption efficiency. In both cases, the EID SPR increased linearly with object thickness and showed only slight dependence on kVp, which is consistent with previous investigations [25,29,30,31].

In comparison to an ideal detector with 100% absorption across the entire spectrum, detectors with Si sensors have poor and significantly decreasing absorption efficiency with increasing photon energies. This results in SPR deviation when using a detector with a thin Si sensor (here, 300 μm thickness). This can be described when considering the detector absorption function D(E) in SPR estimation as well as the energy-integration process,

(4) $$SPR=\frac{\int_{TH}^{kVp}E\cdot I_{S}\left(E\right)D\left(E\right)dE}{\int_{TH}^{kVp}E\cdot I_{P}\left(E\right)D\left(E\right)dE},$$

where $I_{s}\left(E\right)$ and $I_{p}\left(E\right)$ represent scatter and primary intensities, respectively. Deviation between ideal- and finite-thickness Si detectors increases with increasing spectral kVp and object thickness (see Figure 4). This is the result of a higher proportion of lower-energy photons in the absorbed spectrum when using an Si detector that has very poor high-energy-absorption efficiency.

Distinction from the above can be made when considering spectrally resolved imaging with sufficiently narrow energy windows. Referring to Equation (4), in a spectroscopic measurement (as in a PCD), the integration limit shrinks to a very narrow energy bin or even simply single energy, resulting in the SPR to be merely a ratio of $I_{S}\left(E\right)$ and $I_{P}\left(E\right)$, thereby removing the D(E) dependence in the SPR estimates. Therefore, the spectral SPR due to object scatter is expected to be identical for all detector sensor materials and thicknesses, making our following findings on spectral SPR more general.

### 3.2. Energy-Resolved SPR

**Figure 5:**
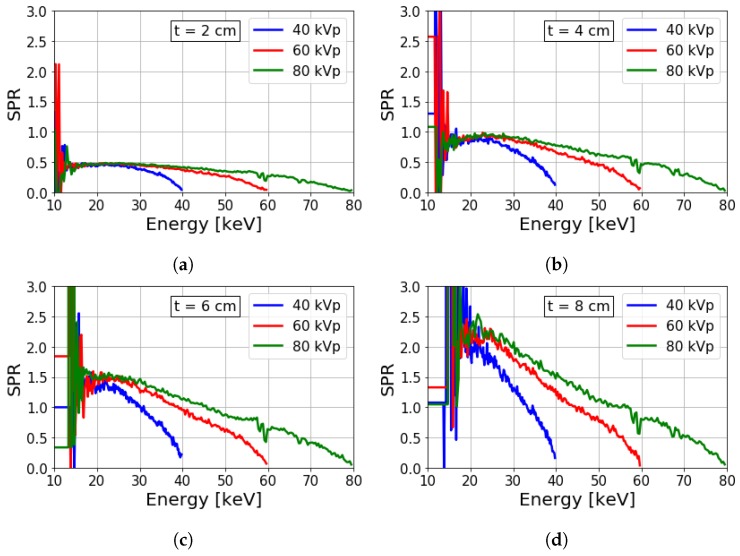
Spectrally-resolved SPRs from simulation using a 0.5 cm air gap for different kVps and object thicknesses of (**a**–**d**) 2, 4, 6, and 8 cm, respectively.

Figure 5 shows energy-resolved SPR values for different kVps and object thicknesses. The SPR remains low near the kVp and increases approximately linearly as the energy is reduced. The elevated SPR at low energies is due to a combination of residual scatter and preferential absorption of the low-energy primary photons. This is highlighted by a rise in SPR with increasing object thickness with decreasing photon energies. Additionally, this causes a low-energy limit where the SPR becomes very large and estimation becomes unreliable due to the eventual high absorption rate and the resulting low photon counts at low energies.

Additionally, Figure 5 includes the spectral SPR for varying kVps. For a given object thickness, increasing the kVp has little effect on the maximum SPR value. This reflects minor kVp dependence on the EID SPRs shown previously in Figure 4. Rather, increasing the kVp reduces the rate at which spectral SPR decreases with energy. For a given energy, the number of incident photons that can contribute to scatter intensity increases with the kVp, which in turn increases the SPR. Convergence of the spectral SPR at low energies may suggest an ‘upper limit’ for the given sample thickness and air gap. However, this assumption may not be suitable for conditions where increased multiple scatter is expected, such as imaging thicker samples. This is partially illustrated by the higher degree of convergence of the 2.0 cm thick sample relative to the 8.0 cm thick sample.

**Figure 6:**
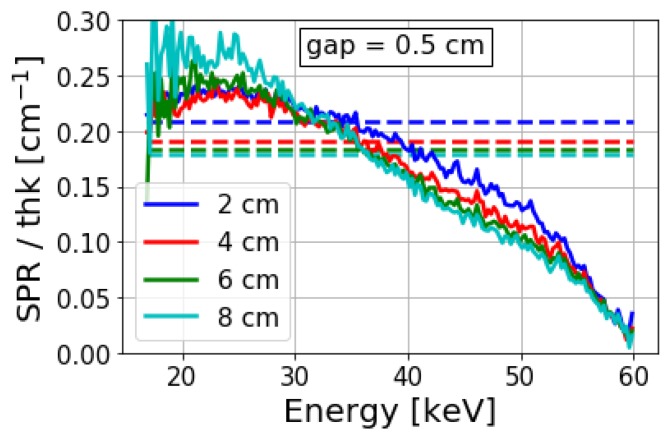
Simulated spectral SPR (solid) and EID SPR (dashed) for varying object thicknesses at a 0.5 cm air gap for a 60 kVp X-ray spectrum. SPR values were normalized by the object thickness to emphasize the linear dependence. EID SPR values lack energy resolution and are instead shown by horizontal lines across the full energy range for comparison with spectral SPR.

Figure 6 shows the EID and spectral SPR for varying object thickness for a small air gap. These SPR values have been normalized by the object thickness to emphasize the relative variations of these two quantities. In each case, the EID SPR lies within the range of spectral SPR values. A small range of lower energies have greater SPR than the EID SPR, while a larger range of energies have lower SPR in comparison to the EID SPR. This implies that quantitative inaccuracies and image quality can be improved over a conventional EID by carefully selecting energy bins. A larger range of energies with SPR lower than the EID SPR can be obtained by increasing the kVp. This is also considering the previous observation that the EID SPR varied only slightly, and that the spectral SPR values were extended across a wider energy range.

### 3.3. SPR Influence on Estimated Linear Attenuation

X-ray scatter reduces apparent sample attenuation by increasing photon counts across the detector. The percent error of the estimated attenuation coefficient can be expressed as

(5) $$\%Error\left(E\right)=\frac{1}{\mu {\left(E\right)}^{ideal}t}\ln\left(\frac{1}{1+\frac{I_{S}\left(E\right)}{I_{P}\left(E\right)}}\right).$$

(see Appendix A for further detail). This expression of the percent error highlights the influence of ideal object attenuation and thickness. The percent error is reduced when $\mu^{ideal}$ is large, which occurs at lower energies. Similarly, the percent error reduces as the object thickness increases. This inverse dependence of these terms reflects the difficulty of identifying objects that have smaller μt values.

**Figure 7:**
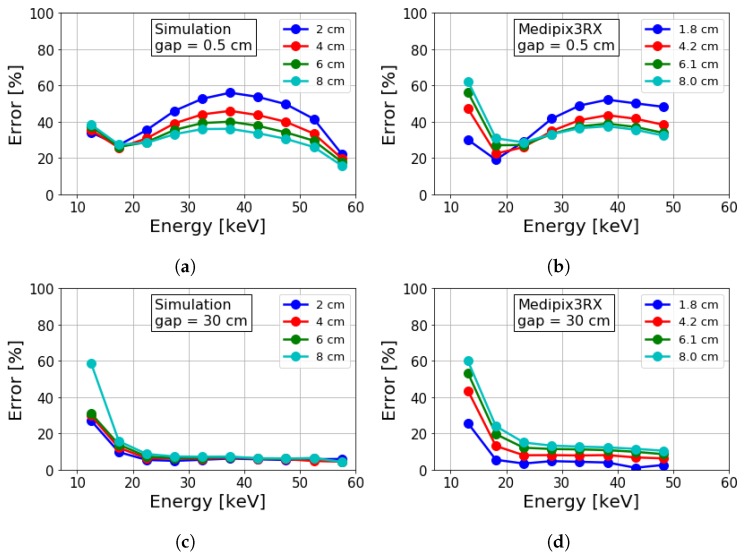
Percent error of estimated attenuation coefficient via simulations (left column) and experiments (right column) for varying energies. Shown for different object thicknesses and air gaps of (**a**,**b**) 0.5 and (**c**,**d**) 30 cm, respectively

Figure 7 shows percent errors of estimated attenuation coefficients for different PMMA thicknesses and air gaps. The error was quite large (>20%) for most energy bins at the small (0.5 cm) air gap. Similar to the spectral SPR (see Figure 6), the error diminished for energies near the kVp. However, the error crested at an intermediate energy bin and began to reduce until a rapid increase at a low energy limit. The error improved at the intermediate energies considering that object attenuation increased as energy was reduced. The rapid increase of errors at low energies was due to a combination of increased scatter and the starvation of primary photons due to higher absorption of the object (PMMA) at these energies.

It was also observed that attenuation errors due to the presence of scatter (see Figure 7a,b) were higher for thinner objects. This can be due to the percent error for estimated attenuation being inversely proportional to the product of object thickness and ideal attenuation. This reflects the concept of a thinner, less attenuating object being less likely to be identified than thicker objects as scatter increases. Examples of these are detecting microcalcifications or small low-contrast cancerous masses in mammograms as well as the detection of explosives among plastics.

Increasing the air gap between object and detector is an effective technique to reduce the number of scattered photons from reaching the detector, and to improve the estimated attenuation accuracy (compare top and bottom Figure 7 rows). However, even in this case, if the detector’s spectral response has some fluence dependence, one can observe a residual error in the attenuation estimation (Figure 7d). This is due to the increasing difference between object intensity and flat-field intensity (measured without the object) in attenuation estimation (Equation (1)), when object thickness increases. These fluence-dependent detector distortions were not accounted for in simulations where these thickness-dependent errors were not observed (Figure 7c). Increasing the air gap from 0.5 to 30 cm reduced the maximum error in attenuation estimation of the 2 cm thick PMMA slab from ∼60% to ∼10%. However, increasing the air gap did not improve the errors at energies <20 keV where the primary was severely reduced by increased object absorption. Additionally, increasing the air gap is also not a practical possibility in many applications, such as in mammography. In these cases, efficient scatter correction is the only solution.

## 4. Discussion and Conclusions

In conclusion, we illustrated several key spectral characteristics of scattered X-ray photons when using an ideal detector and a silicon detector. These general characteristics can be applied to any PCD. For high-Z sensors like CdTe and CdZnTe, there are additional detector distortions to consider and for which to correct . Our finding that a large number of high-energy bins in PCD measurement exhibit lower SPR than a conventional EID X-ray detector, used clinically and industrially, can offer the means to effectively use these detectors without physical antiscatter grids. The spectral characteristics we reported could also be used for developing design and algorithmic strategies for energy-weighting, and material-decomposition and -characterization methods for which these detectors are being developed.

## Appendix A

The following information has been included to provide additional detail to the percent errors described in Section 3.3. Although the SPR continuously increased as energy was reduced (see Figure 6), the percent error of the attenuation may not follow a similar trend due to dependence on object attenuation and thickness. The measured attenuation coefficient could be described by [23]:

(A1) $$\begin{array}{cc} \mu (E)t & =\ln\left(\frac{I_{0}\left(E\right)}{I_{P}\left(E\right)+I_{S}\left(E\right)}\right)\\ \; & =\ln\left(\frac{I_{0}\left(E\right)}{I_{P}\left(E\right)}\cdot \frac{1}{1+\frac{I_{S}\left(E\right)}{I_{P}\left(E\right)}}\right)\\ \; & =\mu {\left(E\right)}^{ideal}t+\ln\left(\frac{1}{1+\frac{I_{S}\left(E\right)}{I_{P}\left(E\right)}}\right). \end{array}$$

By combining Equations (2) and (A1), the percent error of the attenuation is described by

$$\%Error\left(E\right)=\frac{1}{\mu {\left(E\right)}^{ideal}t}\ln\left(\frac{1}{1+\frac{I_{S}\left(E\right)}{I_{P}\left(E\right)}}\right).$$

The inverse relationship on object properties suggests that accuracy improves at lower energies (where μ(E) is elevated) and as thickness increases. Figure 7a,b reflects these considerations.
